# Associations Between Alzheimer’s Disease and Rett Syndrome: Clinical Trials of NA‐831 for the Treatment Alzheimer’s and NA‐921 for Rett Syndrome

**DOI:** 10.1002/alz70861_108250

**Published:** 2025-12-23

**Authors:** Lloyd Tran, Fern Vu, Zung Vu Tran

**Affiliations:** ^1^ Biomed Industries, Inc., San Jose, CA USA; ^2^ MedAware Systems, Inc., Broomfield, CO USA

## Abstract

**Background:**

Alzheimer's disease (AD) and Rett syndrome (Rett) share overlapping genetic and molecular features. NA‐831, a small‐molecule drug, promotes neuroprotection and neurogenesis for AD treatment. NA‐921, an analog of NA‐831, modulates MeCP2 expression and function for Rett treatment.

**Method:**

NA‐831: A randomized clinical trial was conducted in 112 participants with mild to moderate AD. Subjects were randomized to receive either NA‐831 or placebo. Patients with mild cognitive impairment (MCI) received 10 mg/day of NA‐831 or placebo orally, while patients with mild to moderate AD received 30 mg/day. Subjects with MCI to meet the NIA‐AA core clinical criteria, CDR score of 0.5 and a Memory Box score of 0.5 or greater at Screening and Baseline and an MMSE ≥22. Subjects with mild to moderate AD had MMSE scores between 17–21. (ClinicalTrials.gov ID: NCT03538522)

NA‐921: A randomized, double‐blind, placebo‐controlled Phase 2/3 study evaluated NA‐921 (Bionetide) in girls and women with Rett syndrome. (ClinicalTrials.gov ID: NCT06849973)

**Result:**

NA‐831: After 24 weeks of treatment, patients with mild to moderate AD receiving NA‐831 showed a significant improvement in ADAS‐Cog‐13 scores, with an average change of 4.1 compared to placebo (p = 0.001; ITT). CIBIC‐Plus results showed improvement in 78% of patients (p = 0.01; ITT). NA‐831 was well tolerated at 30 mg/day, with no serious adverse events observed, suggesting a safer oral alternative to current AD treatments.

NA‐921: NA‐921 significantly improved outcomes in Rett syndrome. On the Rett Syndrome Behavior Questionnaire (RSBQ), the least squares mean change at week 12 was ‐5.5 for NA‐921 vs. ‐1.6 for placebo (p = 0.001; n = 86 vs. 87). For Clinical Global Impression–Improvement (CGI‐I), scores at week 12 were 3.60 (NA‐921) vs. 3.83 (placebo), with a significant effect size of 0.42 (p = 0.0020). NA‐921 is a potential treatment for Rett Syndrome with fewer side effects and improved patient retention rates.

**Conclusion:**

These findings support a potential mechanistic link between Alzheimer’s disease and Rett syndrome. Both NA‐831 and NA‐921 show promise as novel, well‐tolerated therapeutic agents. Further studies are warranted to validate these results and deepen our understanding of the shared molecular pathways between this neurodegenerative and neurodevelopmental disorder.

